# Pregnancy mobile app use: A survey of health information practices and quality awareness among pregnant women in Australia

**DOI:** 10.1177/17455057241281236

**Published:** 2024-11-05

**Authors:** Bonnie R Brammall, Melanie J Hayman, Cheryce L Harrison

**Affiliations:** 1 Monash Centre for Health Research and Implementation, School of Public Health and Preventive Medicine, Monash University, Clayton, VIC, Australia; 2 Department of Diabetes and Vascular Medicine, Monash Health, Clayton, VIC, Australia; 3 Appleton Institute, School of Health, Medical and Applied Sciences, Central Queensland University, Rockhampton, QLD, Australia

**Keywords:** digital health, pregnancy, smartphone apps, mobile phone, health literacy

## Abstract

**Background:**

Health-related mobile applications (apps) have the potential to improve health knowledge and promote healthy behaviours during pregnancy. Pregnancy apps are popular and extensively used by consumers.

**Objective:**

This study investigates the usage patterns, decision-making criteria and concerns regarding the quality and credibility of health-related information within pregnancy mobile applications. The aim of this study is to understand consumer perspectives to potentially contribute to guidelines for apps containing health-related information.

**Design:**

A cross-sectional study, utilising an online questionnaire for data collection.

**Methods:**

The study surveyed pregnant women in Australia who were recruited via online platforms, including social media and paid Facebook ads. Participants completed a 29-item questionnaire assessing their use of pregnancy apps, sources of health information and perceptions of app quality and safety, with data collected and analysed using the Qualtrics platform and SPSS Statistics.

**Results:**

The survey was survey completed by 427 current-or-recently pregnant individuals, aged 18 or over and located in Australia. Overall, 62.3% were currently pregnant and 37.7% were recently pregnant, within 6 months. Medical practitioners were the primary source of pregnancy-related health information, and pregnancy apps were the third most common source. Pregnancy apps were considered to be a trustworthy source of information by 63.8% of respondents and the majority used apps during pregnancy (94.2%). Of those who used pregnancy apps (*n* = 325), information being safe and trustworthy was their top priority when selecting an app. However, 35.5% (*n* = 115) had encountered information in an app they felt was unsafe or conflicted with previous knowledge or advice. Only 4.6% (*n* = 15) were aware that health-related apps are not screened for accurate information/undergo quality assurance checks before being made available to download. If provided with a guide to evaluate app quality, 74.6% (*n* = 241) would utilise the tool.

**Conclusions:**

These findings highlight a need to promote the critical assessment of health information within pregnancy apps and to develop resources to support consumers in doing so.

## Introduction

The use of pregnancy mobile applications (apps) has gained considerable popularity among expectant parents, becoming an integral part of modern pregnancy information provision and support.^1^ A plethora of apps focussed on pregnancy health (e.g. healthy diet, physical activity and weight), self-monitoring (e.g. kick counters and contraction timers), conditions (e.g. gestational diabetes mellites and pre-eclampsia) and peer support are available, with embedded tools and resources. As many individuals commonly search online for health information before meeting or consulting with a medical practitioner,^2^ there is increasing concern for those who consider information on the internet as safe and useful, without health professional input or validation.^1^

It is estimated that 65% of the global population are now using the internet, and the majority (95%) of internet users do so through mobile phones (i.e. smartphones), accounting for ~60% of global web traffic.^3^ Australia’s internet penetration rate exceeded 96% of the total population at the start of 2023,^4^ with the rate of smartphone ownership projected to reach 87% by 2026,^5^ undoubtably contributing to an associated surge in app use. During pregnancy, this offers a convenient way for expectant parents to access information and resources^6,7^; however, it is crucial to consider the quality and accuracy of the information they provide. Considerable research has been invested to assess the quality of apps, with many reviews in pregnancy reporting suboptimal quality, inaccurate information, limited ability to modify behaviour and failure to alert women to seek health professional advice if warranted.^7–21^ Yet, the translation of these limitations to end users is largely unknown, and there is a paucity of information, resources or screening checklists available to guide consumers towards use of credible apps during pregnancy.^22^ Previous research has largely focussed on reasons for consumer engagement during pregnancy^23,24^; however, there remains limited knowledge^6^ on end user perspectives regarding how consumers choose pregnancy apps and how, or if, they consider the quality and safety of information they interact with. Moreover, the surge in app usage during pregnancy coincides with significant investments in developing digital interventions (or mHealth interventions) tailored for expectant mothers.^25–28^ These investments aim to establish a research consensus and evidence-based support framework for maternal healthcare. However, despite the proliferation of commercially available pregnancy apps directly accessible to consumers, their impact remains largely uncharted beyond clinical or research contexts. To address this gap, this paper aims to gather insights into how individuals access, evaluate and utilise health information during pregnancy, with a specific focus on digital resources such as pregnancy apps and to understand their perceptions regarding the safety and quality of health-related information within apps. Overall, this research seeks to understand user perspectives and self-reported behaviour in order to potentially contribute to guidelines for choosing reliable pregnancy apps or to support the case for better regulation of apps providing health information.

## Materials and methods

### Study design

The study is a cross-sectional study, utilising a questionnaire-based method for data collection. Ethics approval for this study was obtained by the Monash Health Research Ethics Committee for research involving humans (RES-19-0000291A) and all participants provided informed consent prior to participation.

### Setting, recruitment and participants

The study was completed by individuals residing in Australia who were currently pregnant or had given birth or been pregnant within 6 months of participation. Exclusion criteria included not being currently or recently pregnant and being under the age of 18. Participants were recruited via the internet, through paid Facebook advertising, posted via the social media page of the facilitating research centre (Monash Centre for Health Research and Implementation, Monash University, Clayton, VIC, Australia) and associated researcher networks including Instagram, LinkedIn, Twitter and the Baby Centre community forum, Australia. The paid advertisement targeted females, located in Australia, aged 25–41, with interests in pre-defined pregnancy/maternal topics (see Supplemental material 1 for details) to obtain a convenience sample.

Participants opted in to the study, on a voluntary basis, by following an electronic link and completing the anonymous, open web-based questionnaire. The questionnaire was open for responses for 50 days (March to May 2023). The Monash University integrated Qualtrics Insight Platform (Qualtrics, Provo, UT^29^) was used to create and distribute the questionnaire, collect response and store data and reports. Inclusion criteria were minimal, including individuals aged 18 years or over and provision of an estimated due date or delivery date aligning with the pre-defined study design timelines (i.e. currently pregnant or delivery date within 6 months). As an incentive, participants were invited to voluntarily provide an email address to enter a draw to win 1 of 10 AU$50 gift vouchers. No other personal information was collected and all other details were de-identified.

### The questionnaire

The 29-item questionnaire, inclusive of consent and screening questions, was adapted from existing health literacy questionnaires (HLQ)^30^ and informed by previous research evaluating consumer facing pregnancy apps^7,15,31^ to assess women’s app use, risk perception and knowledge and critical assessment of mHealth tools and information (Supplemental material 2) and are described below. The questions were developed in consultation with a multi-disciplinary team across public health, nutrition and exercise physiology; as well as experts in digital health and information technology. The estimated completion time was 10.9 min. To reduce time, number and complexity of the questions, adaptive questioning was used to conditionally display questions based on responses to other items. The survey was user tested by *n* = 2 consumers and analysed for accessibility, errors, compliance and data sensitivity using the Qualtrics Insight Platform, Expert Review function. An overview of the questionnaire, based on the Checklist for Reporting Results of Internet E-Surveys (CHERRIES),^32^ is displayed in Supplemental material 3.

#### Demographics

Age, pregnancy status, employment status, occupation type and highest level of educational attainment and residential post code were collected. Occupation types were informed by the Australian and New Zealand Standard Classification of Occupations,^33^ and a free text option was provided if a participant’s occupation did not align with those provided. Occupation classifications were displayed with major groups and sub-major groups (e.g. professional (perform and apply creative and/or analytical tasks with knowledge and experience in arts or design; business; law; engineering; transport; physical, life or social sciences; health, education or welfare; information technology^33^)). Socio-economic status was estimated according to participant’s postcode, using the deciles in the Australian Socio-Economic Indexes for Areas (SEIFA) Index of Relative Socio-economic Disadvantage.^34^ The index ranks areas based on factors such as income, education, employment and access to services, providing a snapshot of relative disadvantage across the country. Higher-level disadvantage signifies greater socio-economic challenges, while moderate and lower levels indicate varying degrees of disadvantage, with lower being less severe. Deciles 1–3 were classified as higher-level disadvantage, decile 4–7 as moderate-level disadvantage and decile 8–10 as lower-level disadvantage. Regional/remote or urban locality was determined by postcode using the Australian Statistical Geography Standard (ASGS).^35^ The ASGS includes five classes: (1) major cities of Australia, (2) inner regional Australia, (3) outer regional Australia, (4) remote Australia and (5) very remote Australia; combined for analysis to major cities, regional (inner and outer) and remote.

#### Health literacy

Participants were asked health literacy questions to assess their ability to find, evaluate and understand health information.^36^ The HLQ is a 44-item measure that captures the concept of health literacy across 9 distinct domains (measured using one scale per domain). The HLQ domains of appraising information (domain 5) and finding information (domain 8) were used in the present study. There are four response options domain 5: strongly disagree, disagree, agree and strongly agree; and five possible responses, for domain 8: cannot do, very difficult, quite difficult, easy and very easy; see Supplemental material 2. Other HLQ domains were considered out of scope and not applied.

#### Sources of health information during pregnancy

To understand where participants obtain health information during pregnancy, they were asked to rank their top 5 sources from the following options: allied health professional (e.g. physiotherapist/exercise physiologist/dietitian); books; flyers; friends or family; evidence-based internet (e.g. government information, medical websites or peer-reviewed literature); general internet (e.g. information from blogs, websites, forums, videos); medical health practitioner (e.g. obstetrician/medical doctor/midwife); podcasts; and pregnancy apps (e.g. lifestyle and tracking), or ‘other’ (free text option). Participants were then asked how trustworthy they perceive the aforementioned information sources to be on a 5-point Likert scale (where 1 = very untrustworthy and 5 = very trustworthy).

#### Pregnancy apps

Participants were asked if they downloaded apps relating to pregnancy on their phone or other device during pregnancy. Those who responded *no* were asked why, with the following options: I preferred information from other sources; I don’t like using mobile apps; I can’t download apps on my phone or device; I was concerned about the quality and or privacy of information within apps; or other (optional free text entry). Non-app users ended the survey after this question, while those who use apps continued to subsequent questions.

Pregnancy app users were asked what information or tools they have used within a pregnancy app (i.e. those for: information/tracking/foetal and/or maternal development; self-managing a health condition/check symptoms; healthy eating, exercise and/or goal setting; prescriptive exercise (i.e. workouts or exercise plans) or diet (i.e. daily food guides); gestational weight gain trackers; forums or peer group discussions; contraction/kick counter; birth planner; calendars or journals; hospital bag list; pregnancy safety information; breastfeeding tips or other (optional free text entry)), how often they use/used apps relating to pregnancy (i.e. daily; every week or so; every month or so; only once or twice; or, after downloading them I did not use them) and what they considered most important when using a pregnancy app (i.e. ease of use; visual appeal; engaging/interactive features; personalised and/or all-in-one information source; safe and trustworthy; protection of privacy and data; or my doctor supports use).

Following this, participants were asked what makes them likely to trust the information within an app (i.e. recommended by friends or family; recommended by medical professional/practitioner; app rating; app description; consumer reviews; number of downloads/installations; referenced information within app; trialled or tested; disclaimer/terms and conditions; health professionals endorsements; cross-checking information with another trusted source (i.e. gov/health website, book, medical leaflet); the app developer; content writer (author) or none of the above/other).

#### Safety, quality and credibility of health-related apps (consumer perception)

Participants were asked of their decision-making process if in receipt of information in a health-related app they felt was unsafe and/or conflicted with what they believe or have been told/read, as well as if they were concerned about the quality or credibility of information within health apps; if they believe that health-related apps have been checked for accurate information/undergo quality assurance checks before being made available to download; and if they would be likely to use a guide on how to accurately ensure a health app is trustworthy.

### Statistical analysis

Data analysis was performed using IBM SPSS Statistics version 27 (Armonk, New York, NY, USA). Descriptive statistics were tested for skewness by using the Shapiro–Wilk test and are presented as mean and standard deviation for normally distributed continuous variables. Frequencies and percentages are presented for categorical variables. For ranking questions, the top 3 choices were used to define preferences for analysis. Comparisons are made to relevant Australian national data in the results sections to define the population compared to national exemplars. All data are presented as mean (±standard deviation [SD]), unless otherwise stated.

## Results

### Demographic characteristics

Paid Facebook advertisements reached 34,896 individuals, and there were 635 post engagements. Reach refers to the total number of unique users who saw the advertisement at least once, and engagement refers to the number of link clicks. In total, 427 consented to participate in the study and 16 did not progress beyond consent. Of those who consented, 62.0% (*n* = 255) were currently pregnant, 37.5% (*n* = 154) had recently given birth or been pregnant within 6 months, and 0.5% (*n* = 2) were neither, and subsequently removed from further analysis as inclusion criteria could not be ascertained. The mean participant age was 32.6 (4.5) years, and 36.0% (*n* = 138) of participants were on maternity leave, while 31.1% (*n* = 119) were employed full-time and 15.9% (*n* = 61) were employed part-time. The majority of participants reported a professional occupation (64.5%, *n* = 240), followed by community and personal service work (16.1%, *n* = 60). There was a high prevalence of participants that were university educated (70.0%, *n* = 268), while ~10% (*n* = 37) completed year 12 or below (Table 1).

**Table 1.** Participant characteristics.

Demographic Characteristics	Mean	SD
Age (years)	33	(4.5)
	Frequency (*n*)	Percentage
Pregnancy status		
Pregnant	255	62.3
No longer pregnant (given birth/been pregnant within the last 6 months)	154	37.7
Total	409	100.0
Area of residence		
Major city	242	63.7
Inner regional	99	26.1
Rural and remote	39	10.3
Total	380	100.0
Australian Socio-Economic Indexes for Areas (SEIFA)		
Higher-level disadvantage	73	19.2
Moderate-level disadvantage	166	55.3
Lower-level disadvantage	141	37.1
Total	380	100.0
Employment status		
Employed full-time	119	31.1
Employed part-time	61	15.9
No paid work (i.e. home duties)	28	7.3
On maternity leave from employer	138	36.0
Paid casual/temporary employment	15	3.9
Unemployed and not seeking employment	4	1.0
Unemployed and seeking employment	5	1.3
Other	13	3.4
Total	383	100.0
Occupation		
Business manager	26	7.0
Professional	240	64.5
Technician/trade worker	18	4.8
Community and personal service worker	60	16.1
Sales	16	4.3
Machinery operators/drivers	1	0.3
Labour	2	0.5
Other	9	2.4
Total	372	100.0
Education		
No schooling completed	2	0.5
Year 10–12, or equivalent (middle or senior high)	35	9.1
Certificate/apprenticeship/trade/technical/vocational training	41	10.7
Diploma/advanced diploma	36	9.4
Bachelor degree or above (university/college)	268	70.0
Prefer not to say	1	0.3
Total	383	100.0

SD: standard deviation.

Compared with key demographic characteristics from available 2021 Australian Census information; a high proportion of participants reported unemployment (9.6%, *n* = 37) compared to Australian females (3.4% unemployed).^37^ We recruited the majority (55.3%, *n* = 166) of our participants from areas of moderate-level disadvantage, and most (63.7%, *n* = 242) resided in major cities of Australia, or inner regional Australia (26.1%, *n* = 99). When compared to national statistics, a smaller proportion of our cohort lived in major city areas (63.7% compared to 72.0%),^38^ with a higher representation of our cohort residing in inner regional (26.1% compared to 18.0%); yet similar proportions of participants residing in rural areas (10.3% compared to 9.9%).^38^

### Health literacy

**Figure 1.**
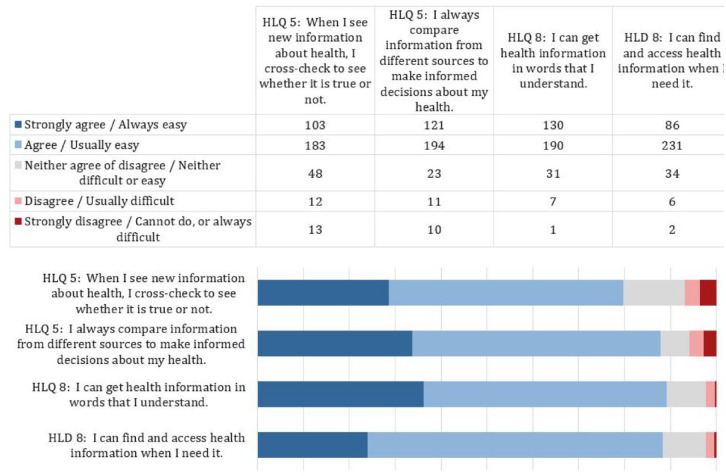
Self-reported health literacy.

Across health literacy domains 5 and 8 of appraising and finding information, the majority of participants reported high levels of health literacy, with ~80%–90% of participants selecting a response demonstrating confidence and ability to assess the quality of and find health information (Figure 1).

### Sources of health information during pregnancy

Participants were asked where they were most likely to source health information during pregnancy. Overall, 53.8% (*n* = 193) reported medical/health practitioners as their primary source of health information during pregnancy, followed by 24.0% (*n* = 86) reporting reputable internet (government information, medical websites or peer-reviewed literature), then 7.8% (*n* = 28) general internet-based information (blogs, websites, forums, videos). When considering participant’s top 3 sources of information, medical practitioners remained the primary source with 82.5% (*n* = 296), followed by reputable internet (73.3%, *n* = 263), then pregnancy apps (36.0%, *n* = 129). Allied health professionals were the fourth most common source of pregnancy health information (32.3%, *n* = 116). Flyers, podcasts and social media were least selected as top 3 sources (0.6%, *n* = 2; 5.8%, *n* = 21; and 9.2%, *n* = 33, respectively; see Table 2).

**Table 2.** Comparison of information source preference and trustworthiness.

Information Source	Ranked preference for health information sources during pregnancy			Trustworthiness of health information sources^a^		
	Primary, *n* (%)	Second, *n* (%)	Third, *n* (%)	Trustworthy, *n* (%)	Neither, *n* (%)	Untrustworthy, *n* (%)
Allied health professional	6 (1.7)	61 (17.0)	48 (13.4)	317 (88.3)	16 (4.5)	1 (0.3)
Books	8 (2.2)	9 (2.5)	20 (5.6)	239 (66.6)	92 (25.6)	6 (1.7)
Flyers	0 (0.0)	0 (0.0)	2 (0.6)	131 (36.5)	151 (42.1)	32 (8.9)
Friends or family	12 (3.3)	21 (5.8)	37 (10.3)	179 (49.9)	142 (39.6)	36 (10.0)
Internet (general)	28 (7.8)	32 (8.9)	50 (13.9)	111(30.9)	173 (48.2)	66 (18.4)
Internet (reputable)	86 (24.0)	105 (29.2)	71 (19.8)	324 (90.3)	23 (6.4)	11 (3.1)
Medical health practitioner	193 (53.8)	73 (20.3)	30 (8.4)	345 (96.1)	10 (2.8)	2 (0.6)
Podcasts	4 (1.1)	6 (1.7)	11 (3.1)	103 (28.7)	191 (53.2)	24 (6.7)
Pregnancy apps	17 (4.7)	38 (10.6)	74 (20.6)	232 (64.6)	105 (29.2)	13 (3.6)
Social media	5 (1.4)	11 (3.1)	16 (4.5)	53 (14.8)	142 (39.6)	155 (43.2)

a Total responses *n* = 359, missing responses for trustworthiness were responses for ‘unsure’.

Trustworthiness of health information sources (Table 2) had similar results to primary sources of information with 96.1% (*n* = 345) considering medical/health practitioners to be trustworthy or very trustworthy; herein referred to as trustworthy. This was followed by reputable internet (90.3%, *n* = 324) and allied health professionals (89.1%, *n* = 320). While books were not a common primary source of information, they were considered to be a trustworthy source by 66.6% (*n* = 239) of participants. Pregnancy apps were considered to be a trustworthy source of information by more than half of the participants (63.8%, *n* = 229). Social media and general internet-based information were deemed the least trustworthy, with 43.2% (*n* = 155) and 18.9% (*n* = 68), respectively, believing these to be untrustworthy or very untrustworthy.

### Pregnancy apps

The majority of participants (94.2%, *n* = 338) used pregnancy apps on their phone or other devices during pregnancy. Of those who did not use pregnancy apps (*n* = 21), 62.0% (*n* = 13) preferred information from other sources, while 19.0% (*n* = 4) don’t like using mobile apps and 19.0% (*n* = 4) were concerned about the quality and/or privacy of information within apps. The cost of ‘reliable’ apps and having had previous pregnancies was also noted as reasons for not using apps. Those who did not use apps were precluded from additional questions about pregnancy apps.

Of those who did use apps (*n* = 325), 30.8% (*n* = 100) used them ‘daily’ and 53.8% (*n* = 175) used them ‘every week or so’. Within apps, information about baby growth and development (91.1%, *n* = 296) and changes to the participant’s body (78.2%, *n* = 254) were the most commonly used app components. The use of these was notably higher than any other component. Forums or group discussions with other women (32.0%, *n* = 104) and pregnancy safety information (28.6%, *n* = 93) were used within apps by some participants. Healthy lifestyle features and self-monitoring tools such as healthy eating and/or exercise information (14.2%, *n* = 46), pregnancy exercise programmes (16.6%, *n* = 54), diet programmes for pregnancy (6.2%, *n* = 20), gestational weight trackers (12%, *n* = 39) and goal setting tools (7.4%, *n* = 24) were infrequently used.

When asked to rank what is most important when using a pregnancy app, first to third choices were combined, which showed 65.5% (*n* = 213) prioritised safe and trustworthy information, followed by easy to understand and find information 53.5% (*n* = 174), and ease of use 52.6% (*n* = 171). Privacy and data protection were reported as a top 3 consideration for 27.4% (*n* = 89). Only 0.9% (*n* = 3) reported their doctor supporting them using it as their most important consideration, which remained the least selected choice when combining first to third (13.8%, *n* = 45).

Participants were then asked what makes them likely to trust the information within an app. More than half of the participants (62.8%, *n* = 204) reported trusting apps that were recommend by their medical practitioner, despite only three participants prioritising their doctor supporting the use of pregnancy apps. Over one-third (38.8%, *n* = 126) of participants advised they checked apps for health professional endorsements as this made them more likely to trust the information within an app. Approximately half of participants, 52.9% (*n* = 172), reported that they cross-check the information in an app with another trusted source (e.g. government/health website, book, medical leaflet). Reading other user reviews and checking the information is referenced increased participants’ trust in an app for 38.5% (*n* = 125). Few participants checked if the app has been trialled or tested (9.8%, *n* = 32), checked who the app developer is (8.0%, *n* = 26) or read the disclaimer/terms and conditions (6.2%, *n* = 20).

### Safety, quality and credibility of health-related apps (consumer perception)

Over one-third (35.5%, *n* = 115) of participants reported finding health information in an app they felt was unsafe and/or conflicted with what they believed or had been told/read; and a further 21.0% (*n* = 68) were unsure if they had. Of those who had encountered unsafe and/or conflicting information, most (48.7%, *n* = 56) reporting engaging with other parts of the app and ignored the information they didn’t agree with. Approximately one-third (36.5%, *n* = 42) cross-checked the information with a trusted source, while some deleted the app (13.9%, *n* = 16). One participant (0.9%, *n* = 1) reported that they followed the information/instructions even though they believed it was unsafe and/or conflicting.

The majority of participants reported remaining neutral (neither unconcerned or concerned) about the quality or credibility of information in apps (48.9%, *n* = 158). More were concerned or very concerned (33.4%, *n* = 108) compared with those who were unconcerned or very unconcerned (17.0%, *n* = 55), while 0.6% (*n* = 2) were unsure. Participants were asked if they believe that apps have been checked for accurate information/undergo quality assurance checks before being made available to download. The majority (95.4% *n* = 308) were unsure or believed they were checked in some capacity (unsure (26.0%, *n* = 84); not very often (41.8%, *n* = 135); most of the time (26.6%, *n* = 86) and all the time (0.9%, *n* = 3)). A very small proportion were aware apps are not regulated/checked for accuracy of content (4.6%, *n* = 15/323).

If provided with a guide or resource on how to accurately assess the credibility of an app, three quarters (74.6%, *n* = 241) said they would be likely to use it. Barriers and enablers for participants using the guide were explored. Most reported that they would be more likely to use the guide if it was free (78.3%, *n* = 253); easy to understand (67.5%, *n* = 218); and quick to use (65.6%, *n* = 212). Being able to save the guide to a phone (digital and not printed) was also considered important to enable use (43.5%, *n* = 141), while being in video format (4.6%, *n* = 15) or having lots of images (10.2%, *n* = 33) was not an influential factor for most and less than a quarter preference detailed information on how to use apps (23.5%, *n* = 76). A small proportion (11.8%, *n* = 38) reported that they would be unlikely to use a guide as they can ‘generally judge if an app is trustworthy’, and reported lack of time or motivation (4.6%, *n* = 15) or interest (2.2%, *n* = 7) as a barrier for using the guide.

## Discussion

Apps can serve as convenient resources for fun and engaging features like tracking the estimated size of a growing baby, observing milestones and connecting with other individuals during pregnancy. However, when they disseminate information that can impact maternal health and decision-making, they should be subject to quality assurance measures and ideally designed in collaboration with healthcare professionals or authorities.^39^ This collaboration can support the provision of up-to-date, safe information that aligns with relevant guidelines. This is particularly crucial given the absence of standardised regulation surrounding pregnancy apps, raising concerns about the reliability of health-related information provided by these apps.^7,31,40,41^ Despite mounting evidence concerning pregnancy app safety, there remains minimal insights from app end users about their perceptions of information quality. Our study assesses how pregnant individuals choose and evaluate apps, and reaffirm their extensive use during pregnancy, and demonstrating that they are generally perceived as trustworthy. Users prioritise design and functionality over factors such as evidence quality, author credentials, disclaimers or trials in assessing app functionality, efficacy, safety, usability or user satisfaction. Popular opinion from reviews or peers largely influences the perceived credibility of these apps. Given that many apps don’t align with current evidence or guidelines, there is concern for potential harm.^31^ This emphasises the need for improved consumer awareness and guidance for credibility assessment to prevent health-related misinformation during pregnancy. Although our sample reported low levels of concern about information quality within pregnancy apps, our findings suggest receptiveness to guidance in assessing app credibility, presenting an opportunity to support accurate information use during pregnancy.

Apps are arrangements of texts, objects and tools that have social and cultural significance.^42^ They draw on, or reproduce knowledge, shared norms and beliefs, including those related to health and risk.^43,44^ While many users believe that health-related content in apps undergoes some form of screening for accuracy or quality assurance, the lack of regulatory oversight presents significant challenges for ensuring consistency and reliability. In this digital health landscape, responsibility for quality assurance typically falls on app developers, and end users are responsible for interpreting and critiquing the information provided. Our study provides insights into users’ trust and critical evaluation of information within pregnancy apps, highlighting their perception of these apps as trustworthy sources of health information. Design-related features such as a clear layout are noted as strong positive factors influencing trust formation of digital information.^45^ However, the focus on aesthetics and functionality among users underscores a potential issue in an unregulated environment, where well-designed apps may convey inaccurate or misleading information.^7,46^ Despite the subjective nature of design assessment, users often prioritise it over considerations of author expertise or evidence-based content, as less than 5% checked who wrote the health information in pregnancy apps. This was comparative to using other consumers’ reviews and number of downloads in impacting trust formation for our cohort. Further to this, the majority of respondents were neither concerned or unconcerned about the quality or credibility of information in apps suggesting consumers’ quality assessment strategies may be suboptimal. While some users cross-check information with other sources, the overall lack of concern about information quality suggests a need for enhanced consumer awareness and guidance in digital health decision-making.

This study revealed that nearly all participants used pregnancy apps, and information about foetal development and tracking of physiological changes during pregnancy remain the most used component, replicating results from a similar Australian survey in 2016.^6^ While 94% of the participants in this study used pregnancy apps, medical practitioners were the primary source of health information for most participants during pregnancy and were also considered the most trustworthy source of information. While medical practitioners are crucial, traditional healthcare has limitations in availability and accessibility. Pregnancy apps can offer convenient information that support healthcare settings; however, at present, their lack of regulation raises concerns about reliability and consistency. In the absence of regulatory measures, empowering consumers to evaluate app quality is vital for informed decision-making and maximising digital health benefits to properly support traditional healthcare. For instance, in our study, pregnancy apps were considered trustworthy sources of information by two-thirds of the participants, yet over a third of those who used pregnancy apps reported encountering unsafe or conflicting information in an app. The inclusion of information that app users considered unsafe or conflicting was not a reason to cease engagement with the app, as almost half continued to engage with other parts of the app, inferring powerful engagement attributes, irrespective of information quality, and/or the ability to discern between reliable and unreliable information. Ensuring a clear distinction between the provision of entertaining apps, social communities and light-hearted facts (such as, ‘your baby is the size of a raspberry’) and critical health and well-being information pertinent to pregnant individuals and their babies is paramount. There is a responsibility to explicitly delineate between these categories to safeguard those who may lack the resources or health literacy to discern between them effectively. Such clarity is essential to prevent misinformation and ensure the well-being of expectant parents and their children.

It is essential that end users are better informed about context of these apps, and effective translation of health information corresponding with the current guidelines and best practice care to minimise potential harm. Australian app regulations are impacted by a complex interplay between developer and consumer considerations; and the involvement of multiple, siloed sectors, crossing medicine, privacy, advertising, finance and content are barriers for regulatory changes that ensure consumer safety.^47^ An Australian review found that the focus tends to be on the commercial loss or gains related to regulation over and above consumer safety, with consumers ultimately assigned as the primary evaluator in selecting safe and credible apps.^47^ As previously recommended, there is a need for resources informing users about app quality, credibility and safety; in a reliable, easy and transparent way, such as independent certification or endorsements.^7^ Recently, a scoping review of pregnancy apps for self-monitoring proposed a ‘Digital Health Scorecard’.^8^ The proposed scorecard includes technical aspects such as privacy, security and performance; usability, encompassing functionally and design elements; and critically, includes as assessment of clinical quality based on direct evidence and credibility; as well as behaviour change techniques such as goal setting, feedback and monitoring.^8^ The current study demonstrates interest in such resources from a consumer perspective, with three quarters reporting they would be likely to utilise a guide on how to accurately assess the trustworthiness of app content. Ideally this would be a free, digital, quick-to-use and easy-to-understand tool. In the context of the sub-optimal quality assessment strategies used by our cohort, and their lack of concern regarding health information quality, it is important that such tools are provided by a credible health authority. Our results position medical practitioners as the best option to provide this guide as the primary, and most trusted source of pregnancy health information. However, while ~60% of participants reported that they would trust apps their medical practitioner recommended and ~40% checked if an app is endorsed by health professionals, less than 1% reported their doctor supporting their use of an app as their most important consideration when using a pregnancy app. These results suggest there may be a lack of linkage between health-related information within apps and medical practitioners, aligning with previous research showing that many women do not discuss the internet-based pregnancy information they read with their health provider.^1^ Medical practitioners may therefore be unaware of potentially inaccurate information about pregnancy and, critically, should not anticipate individuals will seek advice about app use and the contained health information. Therefore, in the context of the extensive number of pregnancy apps and their widespread use, it is important that medical practitioners and health professionals working with pregnant individuals are aware of apps and provide support or resources to guide app-related choices. Additionally, co-design of digital resources should occur to ensure a balance between the attributes valued by end users of apps, alongside health-information delivered in a way that women value as engaging, trustworthy and safe. Previous research suggests that involving relevant expertise in app development does not compromise user downloads of apps, suggesting that quality can be optimised without compromising popularity,^48^ suggesting benefits for the health sector, commercial app developers and app users.

### Strengths and limitations

To our knowledge, this is the first study that evaluates how women select pregnancy apps and perceive and consider their associated health-information in a representative sample of Australian women, with current or recent lived pregnancy experience. Our questionnaire covered an extensive range of factors assessing app use and selection and health information attainment and assessment, in line with health literacy domains for finding and appraising health information.^30^ While some of our survey instruments were not formally validated or tested, they were carefully constructed by a team of experts in pregnancy, lifestyle, women’s health and digital health platforms. The aim is to gather nuanced insights and diverse perspectives that would not have been captured by standardised tools. It is important to note that our approach seeks to complement validated questionnaires to provide a more comprehensive exploration, as validated instruments may sometimes restrict new exploratory insights. Due to the exploratory nature of the survey, a sample size calculation was not determined.

This study had some limitations. App use and selection may be affected by economic status, education level or other demographics. Individuals with higher economic status may have access to a wider range of apps, including premium or paid apps that may offer more features or more reliable information. While those with lower economic status might be limited to free apps, which may have varying degrees of information accuracy. Additionally, users with higher education levels may possess stronger health literacy skills, enabling them to better evaluate the quality of health information presented in the apps, making them more discerning when it comes to assessing the credibility of the information provided. Other demographic factors, such as age, cultural background or language proficiency, could also influence app selection and how users assess the information quality. Different groups may have specific needs or preferences for the type of information they seek or the design and usability of the apps they prefer. We note reasonable distribution of employment and socio-economic status; however, the majority of our cohort were university educated. In addition, the study used a random sample of women; however, a more diverse representation of health literacy levels would have improved insights for women at higher risk for unsafe information. Finally, our results regarding pregnancy apps are limited in their generalisability to apps for other health domains, but can be used to inform research in these areas.

## Conclusion

Our results demonstrate a need to support women in their selection and assessment of pregnancy apps. App users appear to be more receptive to aesthetic and engagement features, functionally and rating or popularity when selecting pregnancy apps, in line with previous research. Given the lack of industry regulation and increasing concerns surrounding credibiliy of health apps, end users should be better supported to assess app quality. Our results demonstrate an opportunity to develop resources to guide decision-making relating to app quality and trustworthiness of information. Medical practitioners are best positioned to provide women with support and guidance, as the primary and most trusted source of health information during pregnancy. Providing resources that inform or guide pregnant individuals’ choices of pregnancy apps can better support health professionals and align with best practice advice. This approach has the potential to optimise health outcomes and reduce pregnancy-related risks. Future research would benefit from exploring if or how medical practitioners and health professionals advise pregnant individuals about pregnancy apps, and understanding barriers and enablers for recommending appropriate apps.

## Supplemental Material

sj-docx-1-whe-10.1177_17455057241281236 – Supplemental material for Pregnancy mobile app use: A survey of health information practices and quality awareness among pregnant women in Australia Supplemental material, sj-docx-1-whe-10.1177_17455057241281236 for Pregnancy mobile app use: A survey of health information practices and quality awareness among pregnant women in Australia by Bonnie R Brammall, Melanie J Hayman and Cheryce L Harrison in Women’s Health

sj-docx-2-whe-10.1177_17455057241281236 – Supplemental material for Pregnancy mobile app use: A survey of health information practices and quality awareness among pregnant women in Australia Supplemental material, sj-docx-2-whe-10.1177_17455057241281236 for Pregnancy mobile app use: A survey of health information practices and quality awareness among pregnant women in Australia by Bonnie R Brammall, Melanie J Hayman and Cheryce L Harrison in Women’s Health

sj-docx-3-whe-10.1177_17455057241281236 – Supplemental material for Pregnancy mobile app use: A survey of health information practices and quality awareness among pregnant women in Australia Supplemental material, sj-docx-3-whe-10.1177_17455057241281236 for Pregnancy mobile app use: A survey of health information practices and quality awareness among pregnant women in Australia by Bonnie R Brammall, Melanie J Hayman and Cheryce L Harrison in Women’s Health

## Declarations

**Ethics:** Ethics approval for this study was obtained by the Monash Health Research Ethics Committee for research involving humans (RES-19-0000291A).

**Consent to participate:** Informed, written, consent was obtained from all subjects involved in the study, which was collected and stored within the survey data.

**Consent to publish:** All participants have consented to their anonymous data being published.

**Availability of data:** Derived data supporting the findings of this study are available from the corresponding author (CLH) on request.

## References

[1] Sayakhot P Carolan-Olah M. Internet use by pregnant women seeking pregnancy-related information: a systematic review. BMC Pregnancy Childbirth 2016; 16(1): 1–10. 27021727 10.1186/s12884-016-0856-5 PMC4810511

[2] Hesse BW Nelson DE Kreps GL , et al. Trust and sources of health information: the impact of the Internet and its implications for health care providers: findings from the first Health Information National Trends Survey. Arch Intern Med 2005; 165(22): 2618–2624. 16344419 10.1001/archinte.165.22.2618

[3] DataReportal. Digital around the world. [Cited 16 June 2023], https://datareportal.com/global-digital-overview (2023, accessed 18 September 2024).

[4] Datareportal. Digital 2023: Australia. [Cited 27 January 2024], https://datareportal.com/reports/digital-2023-australia#:~:text=Australia’s%20internet%20penetration%20rate%20stood,percent)%20between%202022%20and%202023 (2023).

[5] Statista. Smartphone penetration rate as share of the population in Australia in 2017 with an estimate until 2026. [Cited 19 June 2023], https://www.statista.com/statistics/321477/smartphone-user-penetration-in-australia/ (2023).

[6] Lupton D Pedersen S. An Australian survey of women’s use of pregnancy and parenting apps. Women Birth 2016; 29(4): 368–375. 26874938 10.1016/j.wombi.2016.01.008

[7] Brammall BR Garad RM Boyle JA , et al. Assessing the content and quality of digital tools for managing gestational weight gain: systematic search and evaluation. J Med Internet Res 2022; 24(11): e37552. 10.2196/37552 PMC9736757 36427237

[bibr8-17455057241281236] Lazarevic N Lecoq M Bœhm C , et al. Pregnancy apps for self-monitoring: scoping review of the most popular global apps available in Australia. Int J Environ Res Public Health 2023; 20(2): 1012. 36673768 10.3390/ijerph20021012 PMC9858738

[bibr9-17455057241281236] Brown HM Bucher T Collins CE , et al. A review of pregnancy apps freely available in the Google Play Store. Health Promot J Austr 2020; 31(3): 340–342. 31225924 10.1002/hpja.270

[bibr10-17455057241281236] Frid G Bogaert K Chen KT. Mobile health apps for pregnant women: systematic search, evaluation, and analysis of features. J Med Internet Res 2021; 23(10): e25667. 10.2196/25667 PMC8561408 34524100

[bibr11-17455057241281236] Gomes MLdS Rodrigues IR Moura NdS , et al. Evaluation of mobile Apps for health promotion of pregnant women with preeclampsia. Acta Paul Enferm 2019; 32: 275–281.

[bibr12-17455057241281236] Lee M Lee H Kim Y , et al. Mobile app-based health promotion programs: a systematic review of the literature. Int J Environ Res Public Health 2018; 15(12): 2838. 30551555 10.3390/ijerph15122838 PMC6313530

[bibr13-17455057241281236] Brunelli L De Vita C Cenedese F , et al. Gaps and future challenges of Italian apps for pregnancy and postnatal care: systematic search on app stores. J Med Internet Res 2021; 23(8): e29151. 10.2196/29151 PMC8386367 34383668

[bibr14-17455057241281236] Dahl AA Dunn CG Boutté AK , et al. Mobilizing mHealth for moms: a review of mobile apps for tracking gestational weight gain. J Technol Behav Sci 2018; 3: 32–40.

[bibr15-17455057241281236] Hayman MJ Alfrey K-L Waters K , et al. Evaluating evidence-based content, features of exercise instruction, and expert involvement in physical activity apps for pregnant women: systematic search and content analysis. JMIR Mhealth Uhealth 2022; 10(1): e31607. 10.2196/31607 PMC8811692 35044318

[bibr16-17455057241281236] Evans K Donelan J Rennick-Egglestone S , et al. Review of mobile apps for women with anxiety in pregnancy: maternity care professionals’ guide to locating and assessing anxiety apps. J Med Internet Res 2022; 24(3): e31831. 10.2196/31831 PMC8987965 35319482

[bibr17-17455057241281236] Faessen JP Lucassen DA Buso ME , et al. Eating for 2: a systematic review of dutch app stores for apps promoting a healthy diet during pregnancy. Curr Dev Nutr 2022; 6(6): nzac087. 10.1093/cdn/nzac087 PMC9197571 35711572

[bibr18-17455057241281236] Brown HM Bucher T Collins CE , et al. A review of pregnancy iPhone apps assessing their quality, inclusion of behaviour change techniques, and nutrition information. Matern Child Nutr 2019; 15(3): e12768. 10.1111/mcn.12768 PMC7650606 30569549

[bibr19-17455057241281236] Tinius RA Polston M Bradshaw H , et al. An assessment of mobile applications designed to address physical activity during pregnancy and postpartum. Int J Exerc Sci 2021; 14(7): 382. 34055180 10.70252/AQIG9215 PMC8136604

[bibr20-17455057241281236] Hayman M Alfrey K-L Cannon S , et al. Quality, features, and presence of behavior change techniques in mobile apps designed to improve physical activity in pregnant women: systematic search and content analysis. JMIR Mhealth Uhealth 2021; 9(4): e23649. 10.2196/23649 PMC8060865 33825693

[21] Overdijkink SB Velu AV Rosman AN , et al. The usability and effectiveness of mobile health technology–based lifestyle and medical intervention apps supporting health care during pregnancy: systematic review. JMIR Mhealth Uhealth 2018; 6(4): e8834. 10.2196/mhealth.8834 PMC5941088 29691216

[22] Lazarevic N Lecoq M Bœhm C , et al. Pregnancy apps for self-monitoring: scoping review of the most popular global apps available in australia. Int J Environ Res Public Health 2023; 20(2): 1012. 36673768 10.3390/ijerph20021012 PMC9858738

[23] Lupton D. The use and value of digital media for information about pregnancy and early motherhood: a focus group study. BMC Pregnancy Childbirth 2016; 16(1): 171. 27435182 10.1186/s12884-016-0971-3 PMC4950377

[24] Lupton D. ‘It just gives me a bit of peace of mind’: Australian women’s use of digital media for pregnancy and early motherhood. Societies 2017; 7(3): 25.

[25] Rhodes A Smith AD Chadwick P , et al. Exclusively digital health interventions targeting diet, physical activity, and weight gain in pregnant women: systematic review and meta-analysis. JMIR Mhealth Uhealth 2020; 8(7): e18255. 10.2196/18255 PMC7382015 32673251

[bibr26-17455057241281236] Oh SS Moon JY Chon D , et al. Effectiveness of digital interventions for preventing alcohol consumption in pregnancy: systematic review and meta-analysis. J Med Internet Res 2022; 24(4): e35554. 10.2196/35554 PMC9039809 35404257

[bibr27-17455057241281236] Hussain T Smith P Yee LM. Mobile phone–based behavioral interventions in pregnancy to promote maternal and fetal health in high-income countries: systematic review. JMIR Mhealth Uhealth 2020; 8(5): e15111. 10.2196/15111 PMC7290451 32463373

[28] Leblalta B Kebaili H Sim R , et al. Digital health interventions for gestational diabetes mellitus: a systematic review and meta-analysis of randomised controlled trials. PLoS Digit Health 2022; 1(2): e0000015. 10.1371/journal.pdig.0000015 PMC9931335 36812531

[29] Qualtrics. Qualtrics. Provo, UT, USA [updated 2023; cited 1 May 2023], https://www.qualtrics.com/ (2023, accessed 18 September 2024).

[30] Australian Institute of Health and Welfare. Health literacy. Canberra: AIHW, 2022.

[31] Carrandi A Hayman M Harrison CL. Safety considerations for assessing the quality of apps used during pregnancy: a scoping review. Digit Health 2023; 9: 20552076231198683. 37675058 10.1177/20552076231198683 PMC10478559

[32] Eysenbach G. Improving the quality of Web surveys: the Checklist for Reporting Results of Internet E-Surveys (CHERRIES).Toronto, Canada: Gunther Eysenbach Centre for Global eHealth Innovation, 2004. 10.2196/jmir.6.3.e34 PMC1550605 15471760

[33] Australian Bureau of Statistics. Classification structure. Canberra: ABS [cited 21 February 2024]. https://www.abs.gov.au/statistics/classifications/anzsco-australian-and-new-zealand-standard-classification-occupations/latest-release (2022, accessed 18 September 2024).

[34] Australian Bureau of Statistics. Socio-Economic Indexes for Areas (SEIFA), Australia. Canberra: ABS [cited 26 June 2023], https://www.abs.gov.au/statistics/people/people-and-communities/socio-economic-indexes-areas-seifa-australia/latest-release (2021).

[35] Australian Bureau of Statistics. Remoteness areas. Canberra: ABS [cited 26 June 2023], https://www.abs.gov.au/statistics/standards/australian-statistical-geography-standard-asgs-edition-3/jul2021-jun2026/remoteness-structure/remoteness-areas (July 2021–June 2026).

[36] Osborne RH Batterham RW Elsworth GR , et al. The grounded psychometric development and initial validation of the Health Literacy Questionnaire (HLQ). BMC Public Health 2013; 13: 658. 23855504 10.1186/1471-2458-13-658 PMC3718659

[37] Australian Bureau of Statistics. Labour force, Australia. Canberra: ABS [cited 20 July 2023], https://www.abs.gov.au/statistics/labour/employment-and-unemployment/labour-force-australia/jun-2023 (2023, accessed 18 September 2024).

[38] Australian Institute of Health and Welfare. Rural and remote health. Canberra: AIHW, 2022.

[39] Lee Y Moon M. Utilization and content evaluation of mobile applications for pregnancy, birth, and child care. Healthc Inform Res 2016; 22(2): 73–80. 27200216 10.4258/hir.2016.22.2.73 PMC4871848

[40] Tripp N Hainey K Liu A , et al. An emerging model of maternity care: smartphone, midwife, doctor? Women Birth 2014; 27(1): 64–67. 24295598 10.1016/j.wombi.2013.11.001

[41] Musgrave LM Kizirian NV Homer CS , et al. Mobile phone apps in Australia for improving pregnancy outcomes: systematic search on app stores. JMIR Mhealth Uhealth 2020; 8(11): e22340. 10.2196/22340 PMC7704277 33196454

[42] Thomas GM Lupton D. Threats and thrills: pregnancy apps, risk and consumption. Health Risk Soc 2016; 17(7–8): 495–509.

[43] Lupton D. Critical perspectives on digital health technologies. Sociol Compass 2014; 8(12): 1344–1359.

[44] Lupton D Jutel A. ‘It’s like having a physician in your pocket!’A critical analysis of self-diagnosis smartphone apps. Soc Sci Med 2015; 133: 128–135. 25864149 10.1016/j.socscimed.2015.04.004

[45] Sbaffi L Rowley J. Trust and credibility in web-based health information: a review and agenda for future research. J Med Internet Res 2017; 19(6): e218. 10.2196/jmir.7579 PMC5495972 28630033

[46] Akbar S Coiera E Magrabi F. Safety concerns with consumer-facing mobile health applications and their consequences: a scoping review. J Am Med Inform Assoc 2020; 27(2): 330–340. 31599936 10.1093/jamia/ocz175 PMC7025360

[47] Parker L Bero L Gillies D , et al. The “Hot Potato” of mental health app regulation: a critical case study of the australian policy arena. Int J Health Policy Manag 2019; 8(3): 168. 30980633 10.15171/ijhpm.2018.117 PMC6462196

[48] Pereira-Azevedo N Carrasquinho E Cardoso de Oliveira E , et al. MHealth in urology: a review of experts’ involvement in app development. PLoS One 2015; 10(5): e0125547. 10.1371/journal.pone.0125547 PMC4436179 25984916

