# Supplementary material for: Pregnancy mobile app use: A survey of health information practices and quality awareness among pregnant women in Australia
Source: Womens Health (Lond). 2024 Nov 5;20:17455057241281236. doi: 10.1177/17455057241281236 (PMC11539094; doi:10.1177/17455057241281236)
Supplement: sj-docx-1-whe-10.1177_17455057241281236 – Supplemental material for Pregnancy mobile app use: A survey of health information practices and quality awareness among pregnant women in Australia [file sj-docx-1-whe-10.1177_17455057241281236.docx]

Pregnancy App Survey

Survey Flow

Block: Participant Information and Consent (1 Question)

Standard: Eligibility screening (5 Questions)

Branch: New Branch

If

If What is the case for you? None of the above Is Selected

EndSurvey:

Standard: Demographic information (5 Questions)

Standard: Sources of health information (18 Questions)

| Page Break |  |
| --- | --- |

Start of Block: Participant Information and Consent

Introduction You are invited to take part in this research project.

 We are interested in exploring women’s use of health and lifestyle applications (‘apps) during pregnancy. The research project you are being asked to participate in aims to understand how and why women use mobile apps during pregnancy; what drives engagement and how they evaluate the safety of apps they engage with. We would like to better understand women’s perceptions of apps to support women in making safe and healthy information choices during pregnancy.

 We are seeking Australian women (aged 18 years and over) who are currently pregnant; or have been pregnant/given birth within the last 6 months. This project has been initiated by The Monash Centre for Health Research Implementation at Monash University, with support from CQUniversity and the Centre of Research Excellence in Health in Preconception and Pregnancy (CRE HiPP).

 Before you continue, please read the Participant Information and Consent Form (version 1 dated 20/02/2023).

 To participate, you must:
 1. have read the Participant Information and Consent Form
 2. be over the age of 18 years
 3. have given birth/been pregnant within 6 months or be pregnant currently

We invite you to provide your email address to go into the draw to receive one of ten $50 AUD vouchers.

| Page Break |  |
| --- | --- |

End of Block: Participant Information and Consent

Start of Block: Eligibility screening

| 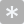 |
| --- |

Consent and confirmation (all three must be confirmed to continue)

- I have read the Participant Information and Consent Form, and wish to participate in this research. (1)
- I am over the age of 18 years. (2)
- I have given birth/been pregnant within the last 6 months or am currently pregnant. (3)

Skip To: End of Survey If Consent != I have read the Participant Information and Consent Form, and wish to participate in this research.

Display This Question:

If Consent = I have read the Participant Information and Consent Form, and wish to participate in this research.

And Consent = I am over the age of 18 years.

And Consent = I have given birth/been pregnant within the last 6 months or am currently pregnant.

| 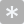 |
| --- |

1 What is your date of birth? (if you are under 18 years of age your response will not be valid)

__

| Page Break |  |
| --- | --- |

Display This Question:

If If What is your date of birth? (if you are under 18 years of age your response will not be valid)&nbsp; Text Response Is Not Empty

2 What is the case for you?

- I have been pregnant/given birth within the last 6 months (1)
- I am currently pregnant (2)
- None of the above (3)

Display This Question:

If 2 = I am currently pregnant

3 What is your estimated due date?

Display This Question:

If 2 = I have been pregnant/given birth within the last 6 months

4 What was your delivery date?

________________________________________________________________

End of Block: Eligibility screening

Start of Block: Demographic information

| Page Break |  |
| --- | --- |

If you would like to enter the draw for a $50 AUD voucher, please provide your email. (optional)

________________________________________________________________

| 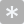 |
| --- |

What is the Australian postcode at your home address?

________________________________________________________________

What is your employment status?

- Employed Full-Time (1)
- Employed Part-Time (2)
- Paid casual / temporary employment (3)
- No paid work (i.e. home duties) (4)
- On maternity leave from employer (5)
- Unemployed and seeking employment (6)
- Unemployed and not seeking employment (7)
- Other (8) __________________________________________________

What is your usual occupation?

- Business Manager (Plan, organise, direct, control, coordinate and review the operations of government, commercial, agricultural, industrial, non-profit and other organisations and departments. For example, CEO, Head of Business, General Manager, Senior Manager) (1)
- Professional (Perform and apply creative and/or analytical tasks with knowledge and experience in arts or design; business; law; engineering; transport; physical, life or social sciences; health, education or welfare; information technology) (2)
- Technician / trade worker (Perform skilled tasks in technical, trade or industry to support sciences, engineering, manufacturing, building) (3)
- Community and personal service worker (Provide services such as hospitality, police and emergency services, security, travel and tourism, fitness and sports. Includes carers in aged care and childcare, those who help health professionals care for patients, those who provide social support and information) (4)
- Sales (Sell and/or manage goods, services and property, and provide sales support) (5)
- Machinery operators / drivers (Operate machines, plant, vehicles and other equipment to perform agricultural, manufacturing and construction functions and move materials) (6)
- Labour (Perform routine and repetitive physical tasks using hand tools, power tools and machines including labourers and those assisting more skilled workers such as trade workers machinery operators and drivers) (7)
- Not applicable (9)
- Other (8) __________________________________________________

What is your highest level of education?

- No schooling completed (1)
- Year 8 or below (Junior High) (2)
- Year 10 or equivalent (Middle High) (3)
- Year 11 or equivalent (4)
- Year 12 or equivalent (Senior High) (5)
- Certificate/apprenticeship/trade/technical/vocational training (6)
- Diploma/advanced diploma (7)
- Bachelor degree or above (university/college) (8)
- Prefer not to say (9)
- Other (10) __________________________________________________

End of Block: Demographic information

Start of Block: Sources of health information

**HLQ (domain 5)**

Which of the following applies to you?

|  | Strongly disagree (1) | Disagree (2) | Neither agree of disagree (3) | Agree (4) | Strongly agree (5) |
| --- | --- | --- | --- | --- | --- |
| When I see new information about health, I cross-check to see whether it is true or not. (1) |  |  |  |  |  |
| I always compare information from different sources to make informed decisions about my health. (2) |  |  |  |  |  |

Which of the following applies to you?

|  | Strongly disagree (1) | Disagree (2) | Neither agree of disagree (3) | Agree (4) | Strongly agree (5) |
| --- | --- | --- | --- | --- | --- |
| When I see new information about health, I cross-check to see whether it is true or not. (1) |  |  |  |  |  |
| I always compare information from different sources to make informed decisions about my health. (2) |  |  |  |  |  |

**HLQ (domain 8)**

Which of the following applies to you?

|  | Cannot do, or always difficult (1) | Usually difficult (2) | Neither difficult or easy (3) | Usually easy (4) | Always easy (5) |
| --- | --- | --- | --- | --- | --- |
| I can get health information in words that I understand. (1) |  |  |  |  |  |
| I can find and access health information when I need it. (2) |  |  |  |  |  |

| 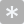 |
| --- |

During pregnancy, where are you most likely to source health information? (rank top 5, most to least; 1 being the most important)

______ Internet (information from blogs, websites, forums, videos) (1)

______ Internet (government information, medical websites or peer-reviewed literature) (2)

______ Pregnancy apps (lifestyle, tracking etc) (3)

______ Social media (e.g. Facebook, Instagram, TikTok) (4)

______ Friends or family (5)

______ Medical health practitioner (obstetrician/medical doctor/midwife) (6)

______ Allied health professional (physiotherapist/exercise physiologist/dietitian etc) (7)

______ Books (8)

______ Flyers (9)

______ Pod-casts (10)

______ Other (11)

How trustworthy do you find the following sources of health information?

|  | Very untrustworthy (1) | Untrustworthy (2) | Neither untrustworthy or trustworthy (3) | Trustworthy (4) | Very trustworthy (5) | Unsure (6) |
| --- | --- | --- | --- | --- | --- | --- |
| Internet (information from blogs, websites, forums, videos) (1) |  |  |  |  |  |  |
| Internet (government information, medical websites or peer-reviewed literature) (2) |  |  |  |  |  |  |
| Pregnancy apps (lifestyle, tracking etc) (3) |  |  |  |  |  |  |
| Social media (e.g. Facebook, Instagram, TikTok) (4) |  |  |  |  |  |  |
| Friends or family (5) |  |  |  |  |  |  |
| Medical health practitioner (OB/medical doctor/midwife) (6) |  |  |  |  |  |  |
| Allied health professional (physiotherapist/Ep/diet etc) (7) |  |  |  |  |  |  |
| Books (8) |  |  |  |  |  |  |
| Flyers (9) |  |  |  |  |  |  |
| Pod-casts (10) |  |  |  |  |  |  |

During pregnancy, did you download apps relating to pregnancy on your phone or other device?

- Yes (1)
- No (2)

Display This Question:

If app use = No

Why not?

- I preferred information from other sources (1)
- I don’t like using mobile apps (2)
- I can’t download apps on my phone or device (3)
- I was concerned about the quality and or privacy of information within apps (4)
- Other (5) __________________________________________________

Display This Question:

If app use = Yes

How often did/do you use apps relating to pregnancy?

- Daily (1)
- Every week or so (2)
- Every month or so (3)
- Only once or twice (4)
- After downloading them I did not use them (5)

Display This Question:

If app use = Yes

What information or tools have you used within pregnancy apps?

- Information/tracking about changes to my body (1)
- Information/tracking about baby growth and development (2)
- Tools to self-manage a health condition/check symptoms (e.g. diabetes in pregnancy, mental health, blood pressure) (3)
- Tools to help me set goals (4)
- Healthy eating and/or exercise information (5)
- Pregnancy exercise programs (i.e. workouts or exercise plans) (6)
- Diet programs for pregnancy (i.e. daily food guides) (7)
- Pregnancy weight gain trackers (8)
- Forums or group discussions with other women (9)
- Contraction counter/timer (10)
- Kick counter (11)
- Birth planner (12)
- Calendar to track prenatal appointments (13)
- Journal for mood and/or symptoms (14)
- Hospital bag list (15)
- Pregnancy safety information (16)
- Breastfeeding tips (17)
- Other (18) __________________________________________________

Display This Question:

If app use = Yes

| 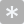 |
| --- |

What is most important to you when using a pregnancy app? (please rank a minimum of 5 responses; 1 being the most important)

______ Easy to use (1)

______ Easy to understand and find information (2)

______ Visually appealing (3)

______ Fun and engaging/interactive features (4)

______ Personalised information to my needs (5)

______ All pregnancy information I need is included (a “one-stop-shop”) (6)

______ Safe and trustworthy information (7)

______ My privacy and data are protected (8)

______ My doctor supports me using it (9)

Display This Question:

If app use = Yes

| 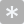 |
| --- |

What makes you most likely to trust the information within an app?

- I trust apps that my friends or family recommend (1)
- I trust apps that my medical professional/practitioner recommend (2)
- I use the app rating (i.e. App store or Google play rating) (3)
- The app description (4)
- I read other peoples’ reviews (5)
- I look at the number of downloads/installations (6)
- I check that information is referenced (7)
- I check if the app has been trialled or tested (8)
- I read the disclaimer/terms and conditions (9)
- I check if the app is endorsed by health professionals (10)
- I cross-check the information I read with another trusted source (e.g. gov/health website, book, medical leaflet) (11)
- I check who made the app (developer) (12)
- I check who wrote the content (author) (13)
- I don’t do any of the above (14)
- Other (15) __________________________________________________

Display This Question:

If app use = Yes

Have you ever come across information in a health-related app you felt was unsafe and/or conflicted with what you believe or have been told/read?

- Yes (1)
- No (2)
- Unsure (3)

Display This Question:

If unsafe info = Yes

What did you do when you came across this information?

- I followed the information/instructions in the app anyway (1)
- I engaged with other parts of the app and ignored the information I didn’t agree with (2)
- I deleted the app (3)
- I cross-checked the information with a trusted source (4)

Display This Question:

If action = I cross-checked the information with a trusted source

Who, or what information source did you cross check with?

________________________________________________________________

Display This Question:

If app use = Yes

Are you concerned at all about the quality or credibility of information within health apps?

- Very unconcerned (1)
- Unconcerned (2)
- Nether unconcerned or concerned (3)
- Concerned (4)
- Very concerned (5)
- Unsure (6)

Display This Question:

If app use = Yes

Do you believe that health-related apps have been checked for accurate information/undergo quality assurance checks before being made available to download?

- Yes, all the time (1)
- Yes, most of the time (2)
- Unsure (3)
- No, not very often (4)
- No, never (5)
- Unconcerned (6)

Display This Question:

If app use = Yes

If you were provided with a guide on how to accurately ensure a health app is trustworthy how likely would you use this?

- Very unlikely (1)
- Unlikely (2)
- Neutral (3)
- Likely (4)
- Very likely (5)
- Unsure (6)

Display This Question:

If app use = Yes

I would be more likely to use a guide to check apps if: (select the response/s that apply to you)

- It was free to use (1)
- It was easy to understand (2)
- It didn’t take long to use (3)
- It was in a video format (4)
- It had lots of pictures (5)
- It had detailed information on how to evaluate apps (6)
- It had good reviews (7)
- It was used by my peers / people I know (8)
- I could save it onto my phone (i.e. a digital guide - not printed) (9)
- I’m unlikely to use a guide, I can generally judge whether an app is trustworthy (10)
- I’m unlikely to use a guide, I don’t have the time / motivation (11)
- I’m unlikely to use a guide, I’m not really interested (12)
- Unsure (13)
- Other (14) __________________________________________________

End of Block: Sources of health information
