# Supplementary material for: Pregnancy mobile app use: A survey of health information practices and quality awareness among pregnant women in Australia
Source: Womens Health (Lond). 2024 Nov 5;20:17455057241281236. doi: 10.1177/17455057241281236 (PMC11539094; doi:10.1177/17455057241281236)

Digital health app use during pregnancy – a consumer perspective

The Investigators are researching women’s use of health and lifestyle applications (‘apps) during pregnancy. The aims of this research are to:

1/ understand how/why women choose mobile apps for health and lifestyle management during pregnancy;

2/ determine if women evaluate the quality and safety of the information in apps they download for use;

3/ scope how we can assist pregnant women in evaluating the quality of information within apps via developed resources (e.g. checklists, factsheets etc);

Recruitment of participants will occur via the investigators established networks and partners. They will use social media using a developed invitation (Appendix 1).

Consent will be obtained via a Qualtrics link with a checkbox for consent.

A quantitative online Qualtrics survey will be used. While participants won’t be reimbursed for their participation, an option to enter a draw for one of 10 $50AUD gift cards will be provided as a token of appreciation.

Appendix 1:

**Invitation text to be used alongside image in Facebook ad, as well as via unpaid posts on social media channels:**

We are interested in exploring women’s use of pregnancy-related health and lifestyle apps.

This survey will help us understand how and why women use mobile apps during pregnancy; what drives use and how women evaluate the safety of apps they engage with. We would like to better understand women’s perceptions of apps to support women in making safe and healthy information choices during pregnancy.

If you would like to be involved please click the link and complete the survey.


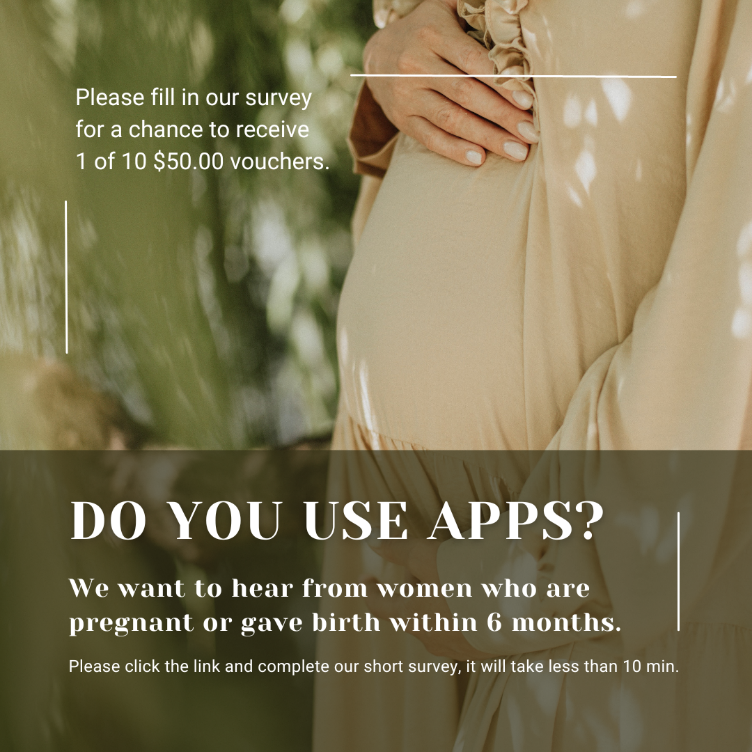


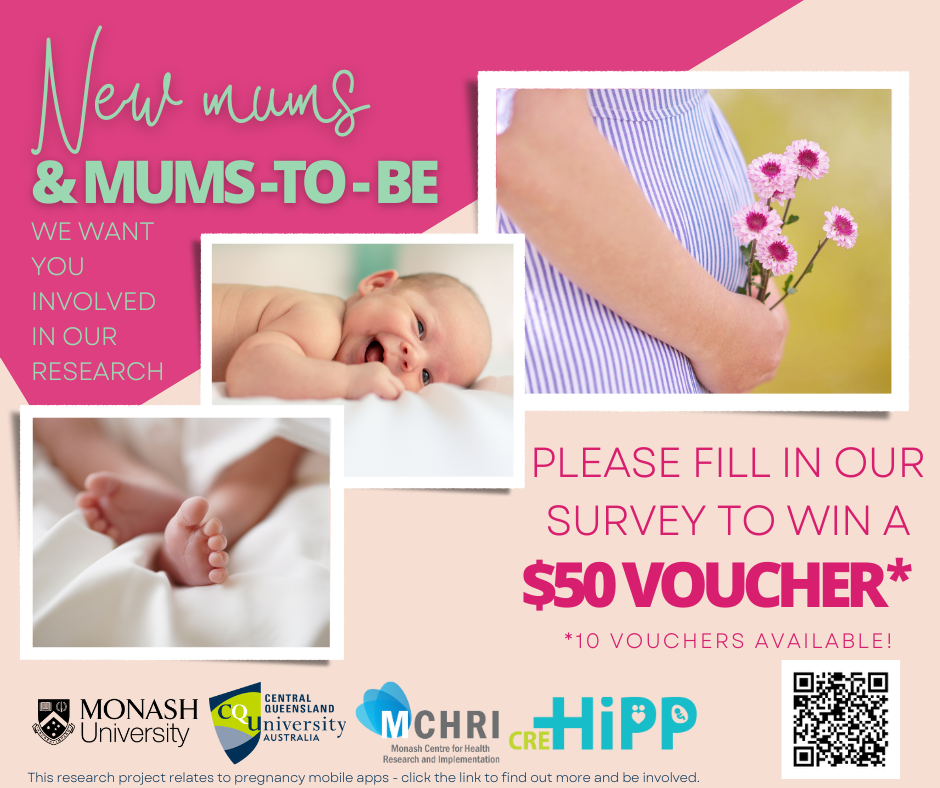


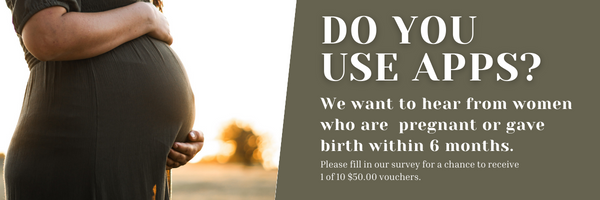

Supplement: sj-docx-2-whe-10.1177_17455057241281236 – Supplemental material for Pregnancy mobile app use: A survey of health information practices and quality awareness among pregnant women in Australia [file sj-docx-2-whe-10.1177_17455057241281236.docx]
