# Supplementary material for: Pregnancy mobile app use: A survey of health information practices and quality awareness among pregnant women in Australia
Source: Womens Health (Lond). 2024 Nov 5;20:17455057241281236. doi: 10.1177/17455057241281236 (PMC11539094; doi:10.1177/17455057241281236)
Supplement: sj-docx-3-whe-10.1177_17455057241281236 – Supplemental material for Pregnancy mobile app use: A survey of health information practices and quality awareness among pregnant women in Australia [file sj-docx-3-whe-10.1177_17455057241281236.docx]

Checklist for Reporting Results of Internet E-Surveys (CHERRIES)

|  | Checklist for Reporting Results of Internet E-Surveys (CHERRIES) | |
| --- | --- | --- |
| Item Category | Checklist Item | Explanation |
| Design |  |  |
|  | Describe survey design | Target population: Individuals residing in Australia who were currently pregnant or had given birth or been pregnant within six months of participation.  Recruitment: Participants were recruited to complete an open survey via the internet. Paid Facebook advertising targeted females, located in Australia, aged 25-41, with interests in predefined pregnancy/maternal topics to obtain a convenience sample. Recruitment posters were also posted to the social media pages and Baby Centre community forum, Australia. |
| IRB (Institutional Review Board) approval and informed consent process |  |  |
|  | IRB approval | Ethics approval for this study was obtained by the Monash Health Research Ethics Committee for research involving humans (RES-19-0000291A). |
|  | Informed consent | Participants were provided with a Participant Information and Consent Form (PCIF) that described the purpose of the survey and length of time. Participants were informed about the length of time their data may be stored for (up to seven years); where the data would be stored (within a secure drive within the School of Public Health and Preventative Medicine at Monash University); contact details were provided for the Principal Investigator; Complaints Contact and Executive Office for the approving Human Research Ethics Committee. |
|  | Data protection | The Monash University integrated Qualtrics Insight Platform (Qualtrics, Provo, UT 24) was used to create and distribute the questionnaire, collect responses and store data and reports. Only those with access to the survey (n=2) could view the data by logging into their Monash University, Qualtrics account. Personal information (email addresses) were not extracted from the integrated platform. |
| Development and pre-testing |  |  |
|  | Development and testing | The survey questions were adapted from existing health literacy questionnaires (HLQ) and informed by previous research evaluating consumer facing pregnancy apps.  The questions were developed in consultation with a multidisciplinary team across public health, nutrition and exercise physiology; as well as experts in digital health and information technology.  The survey was user tested by n=2 consumers, and analysed for accessibility, errors, compliance and data sensitivity using the Qualtrics Insight Platform, Expert Review function. |
| Recruitment process and description of the sample having access to the questionnaire |  |  |
|  | Open survey versus closed survey | The survey was open for completion; access was not restricted via a unique link or password. |
|  | Contact mode | Initial contact with the potential participants was made on the Internet. |
|  | Advertising the survey | The survey was announced via social media. See Supplementary Material 1. |
| Survey administration |  |  |
|  | Web/E-mail | Posted on Web sites: (Facebook, Instagram, X (formally, Twitter), and Baby Centre forum (Australia)). |
|  | Context | Facebook was used as the main recruitment platform. Paid advertisements were directed to two audiences:  Audience 1:  Location - Living in: Australia Age: 25-41  Gender: Female Interests: Pregnancy (mammals); Motherhood; Infant; Bugaboo International; or Due Date  Audience 2:  Location - Living in: Australia Age: 25-41  Gender: Female Interests: Motherhood; Health & wellness; Baby shower; Pregnancy (mammals); Parents (up to 12 months) |
|  | Mandatory/  voluntary | Voluntary survey. |
|  | Incentives | A monetary incentive was offered. Participants could enter the draw to win one of 10 $50 AUD gift vouchers. |
|  | Time/Date | Open for 50 days (Mar-May 2023) |
|  | Randomization of items or questionnaires | Nil. |
|  | Adaptive questioning | Adaptive questioning was used (see Supplementary Material 2 for details). |
|  | Number of Items | Refer to see Supplementary Material 2. |
|  | Number of screens (pages) | Refer to see Supplementary Material 2. |
|  | Completeness check | A completeness check was provided to participants when engaging with the survey, facilitated by the Qualtrics Platform. |
|  | Review step | A back button was provided throughout for participants to review or change their responses. |
| Response rates |  |  |
|  | Unique site visitor | Unique visitors were based on IP addresses. No duplicate IP addresses were identified. |
|  | View rate (Ratio of unique survey visitors/unique site visitors) | Based on data from the paid Facebook advertising; the ad reached n=34,896, the participation rate of those who provided consent (n=427) was 1.2%. However we note that data is not available for those who saw the survey advert in other platforms (Instagram, X, Baby Centre Forum etc) |
|  | Participation rate (Ratio of unique visitors who agreed to participate/unique first survey page visitors) | Based on Facebook engagement (n=635 link clicks), the participation rate of those who provided consent (n=427) was 67.2%. However we note that data is not available for those who viewed the survey from other platforms (Instagram, X, Baby Centre Forum etc) |
|  | Completion rate (Ratio of users who finished the survey/users who agreed to participate) | The attrition rate was 84.5%. |
| Preventing multiple entries from the same individual |  |  |
|  | Cookies used | Nil. |
|  | IP check | IP addresses were screened to identify duplicate entries from the same user. No two entries from the same IP address were submitted. |
|  | Log file analysis | Nil. |
|  | Registration | N/A |
| Analysis |  |  |
|  | Handling of incomplete questionnaires | Relevant information provided in results - demographic data was reported for all participants. All other responses are reported based on the number of responses. Data was only excluded for those who did not progress past consent, and those who were not eligible (n=2, not pregnant or recently given birth). |
|  | Questionnaires submitted with an atypical timestamp | Not considered. |
|  | Statistical correction | Nil. |
